# C-Terminal Substitution of HBV Core Proteins with Those from DHBV Reveals That Arginine-Rich ^167^RRRSQSPRR^175^ Domain Is Critical for HBV Replication

**DOI:** 10.1371/journal.pone.0041087

**Published:** 2012-07-20

**Authors:** Jaesung Jung, Hee-Young Kim, Taeyeung Kim, Bo-Hye Shin, Gil-Soon Park, Sun Park, Yong-Joon Chwae, Ho-Joon Shin, Kyongmin Kim

**Affiliations:** Department of Microbiology, Ajou University School of Medicine, Woncheon-dong, Suwon, Korea; Yonsei University, Republic of Korea

## Abstract

To investigate the contributions of carboxyl-terminal nucleic acid binding domain of HBV core (C) protein for hepatitis B virus (HBV) replication, chimeric HBV C proteins were generated by substituting varying lengths of the carboxyl-terminus of duck hepatitis B virus (DHBV) C protein for the corresponding regions of HBV C protein. All chimeric C proteins formed core particles. A chimeric C protein with 221–262 amino acids of DHBV C protein, in place of 146–185 amino acids of the HBV C protein, supported HBV pregenomic RNA (pgRNA) encapsidation and DNA synthesis: 40% amino acid sequence identity or 45% homology in the nucleic-acid binding domain of HBV C protein was sufficient for pgRNA encapsidation and DNA synthesis, although we predominantly detected spliced DNA. A chimeric C protein with 221–241 and 251–262 amino acids of DHBV C, in place of HBV C 146–166 and 176–185 amino acids, respectively, could rescue full-length DNA synthesis. However, a reciprocal C chimera with 242–250 of DHBV C (^242^R***AG***S***PL***PR***S*** ^250^) introduced in place of 167–175 of HBV C (^167^R***RR***S***QS***PR***R*** ^175^) significantly decreased pgRNA encapsidation and DNA synthesis, and full-length DNA was not detected, demonstrating that the arginine-rich ^167^RRRSQSPRR^175^ domain may be critical for efficient viral replication. Five amino acids differing between viral species (underlined above) were tested for replication rescue; R169 and R175 were found to be important.

## Introduction

Hepadnaviruses are small, enveloped DNA viruses that replicate preferentially in liver cells and are associated with acute and chronic hepatitis, cirrhosis, and hepatocellular carcinoma [1]. Hepatitis B virus (HBV), a prototypic hepadnavirus, has a partially duplex relaxed circular (RC) DNA genome, which replicates by reverse transcription of a pregenomic RNA (pgRNA) to produce genomic DNA.

The core (C) protein of HBV consists of 183 or 185 amino acids that form core particles via dimeric intermediates [2]. Assembly of replication-competent HBV core particles requires interaction of pgRNA with the polymerase (P) and C proteins. The amino-terminus of C protein (amino acids 1–144) participates in core particle assembly through protein-protein interaction and is, by itself, assembly competent [3], [4] The carboxyl-terminus of C protein contains a protamine-like nucleic acid binding domain rich in arginine. Although this region is dispensable for core particle assembly, it is involved in hepadnaviral replication, including pgRNA encapsidation and DNA replication [3]–[10]. The importance of C protein’s carboxyl-terminus in hepadnavirus pgRNA encapsidation and DNA replication has been suggested through experiments with a series of carboxyl-terminal truncation mutants [3]–[6], [9], [10]. The C protein amino-acid 164 variant deficient in 19 carboxyl-terminal amino acids (C164), corresponding to amino-acid 166 in our *adw* subtype, is competent for pgRNA encapsidation, but not for synthesis of full-length RC DNA [6], [9], [10]. DNA synthesized in variant C164 core particles is predominantly spliced [6], [9]. However, a C variant containing 173 amino acids and lacking ten amino acids at the carboxyl-terminus (C173), corresponding to 175 amino acids in our *adw* subtype, was as competent for synthesis of full-length RC DNA as *wild type* (*wt*) C protein, suggesting that nine carboxyl-terminal amino acids (165–173) are sufficient for replication-competence [9]. Although it has been suggested that these residues are important for selective and/or productive viral RNA encapsidation in deletion- and site-directed mutants [9], a direct demonstration of the amino acid residues or motif in the carboxyl-terminus of C protein critical for hepadnavirus pgRNA encapsidation or DNA replication has not yet been performed.

Heterologous complementation to generate chimeric proteins of related viruses is a way of identifying the viral-protein amino acid residues or motifs crucial for replication. Chimeric viruses or proteins have been used to identify viral *cis*-acting sequences and the functions of protein domains [11]–[14]. Chimeras of duck HBV (DHBV) and heron HBV, another avian hepadnavirus sharing 79% nucleotide identity with DHBV, have been used to elucidate the functional interactions between *cis*-acting sequences and viral components for pgRNA encapsidation and plus-strand DNA synthesis [15]–[17]. The genomes of HBV and woodchuck hepatitis virus (WHV) share approximately 60% identity [18], [19]; those of HBV and DHBV share 40% homology [19]. However, heterologous complementation with related hapdnaviruses such as DHBV or WHV cannot be performed; while HBV replication is restored easily by complementation with WHV C and/or P proteins and vice versa, it cannot be complemented at all by DHBV C and/or P proteins and vice versa [20], [21]. Therefore, use of DHBV or WHV chimeric viruses or proteins to complement HBV replication has not been explored.

In the present study, HBV chimeric C proteins were constructed by exchanging portions of the carboxyl-terminus of HBV C protein with the corresponding regions of DHBV C protein, while retaining *wt* HBV C protein amino-terminal sequence to investigate the critical regions for pgRNA encapsidation or HBV DNA synthesis. DHBV C protein, which consists of 262 amino acids, can form a three-dimensional core particle similar in structure to that of HBV [22]. Use of these chimeras demonstrated that some chimeric core particles are replication-competent, complementing HBV C proteins in C-deficient mutants to effect pgRNA encapsidation concomitant with reverse transcription. These results indicate that 40% amino acid sequence identity or 45% homology in the carboxyl-terminus of C protein is sufficient for HBV pgRNA encapsidation and DNA synthesis, even though predominantly spliced HBV DNA was synthesized. Serial substitutions of HBV C protein with the corresponding regions of DHBV C protein further allowed us to demonstrate that residues 167–175, ^167^R***RR***S***QS***PR***R*** ^175^, may be critical for full-length RC DNA synthesis as long as residues from 146–166 maintain 62% homology. Although the importance of residues R167, S170, P173, and R174 in the HBV C protein could not be examined due to the presence of identical residues in the corresponding region of DHBV (^167^R***RR***S***QS***PR***R*** ^175^ in HBV *vs* ^242^R***AG***S***PL***PR***S*** ^250^ in DHBV) in HHDH C chimera, in which HBV C 167–175 was replaced by DHBV C 242–250, we analyzed the importance of R168, R169, Q171, S172, and R175 residues using a series of point mutants. By analyzing the A168R, G169R, P171Q, L172S, and S175R mutants in HHDH C chimera, we further demonstrated that the R169 and R175 residues may be important for HBV replication and that S172 may be important for core particle formation, but not for pgRNA encapsidation or DNA synthesis. The importance of residues 167–175 in HBV C protein for replication in the context of neighboring amino acids or motifs is discussed.

## Results

### Chimeric C Protein Expression and Core Particle Formation

**Figure 1:**
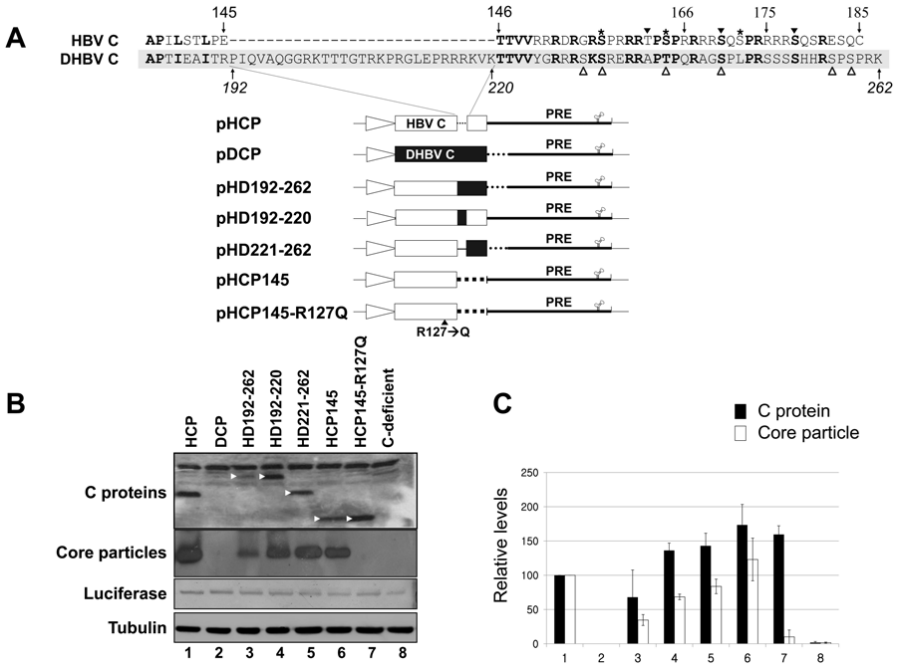
C protein expression and core particle assembly by chimeric C protein variants. (A) Schematic diagrams of HBV, DHBV, and chimeric C protein variant constructs aligned with amino acid sequences of HBV and DHBV C protein carboxyl-terminal domains. Amino acids in bold are identical or homologous. SRPK and PKA phosphorylation sites of HBV are marked with asterisks and arrowheads, respectively. Phosphorylation sites of DHBV [38], [41] are marked with open arrowheads. Amino acid sequences of the HBV and DHBV C proteins are presented as open and closed boxes, respectively. The cytomegalovirus immediate early (CMV IE) promoter is represented as an open arrow. PRE, post-transcriptional regulatory element. (B) Identification of C protein and core particles by chimeric C protein variants. To examine expression of C protein variants, lysates from HuH7 cells transfected with a pHCP, pDCP, pHD192–262, pHD192–220, pHD221–262, pHCP145, pHCP145–R127Q, or C-deficient mutant were electrophoresed on 12% SDS-PAGE gels and protein levels visualized by Western blotting using polyclonal rabbit anti-HBc antibody (top panel). C protein variants (arrowheads) with expected molecular weights are indicated. The C-deficient mutant lacks C protein due to the introduction of a stop codon at Glu 8 in the C ORF. The pHCP and the C-deficient mutant constituted positive and negative controls, respectively. Transfection experiments were repeated four times. To detect core particles formed by C protein variants from native agarose gels, isolated core particles were transferred to PVDF membranes and incubated with polyclonal rabbit anti-HBc antibody (second panel). The Renilla luciferase expression plasmid phRL-CMV was co-transfected into HuH7 cells as a transfection control (third panel). Luciferase and α-tubulin (bottom panel) levels were determined by Western blotting using polyclonal rabbit anti-luciferase and monoclonal mouse anti-tubulin antibodies as transfection and loading controls, respectively. HRP-conjugated secondary antibody and enhanced chemiluminescence were used to visualize C, α-tubulin, and luciferase proteins and core particles. (C) Relative levels of C protein expression and core particle assembly by chimeric C protein variants. Relative levels of C proteins, core particles, and luciferase were measured with the Fujifilm Image Gauge V4.0 program. Relative levels of C protein variant expression and core particle assembly were compared to normalized transfection efficiencies. The data represent the mean ± standard deviation (SD) from four independent experiments.

**Figure 2:**
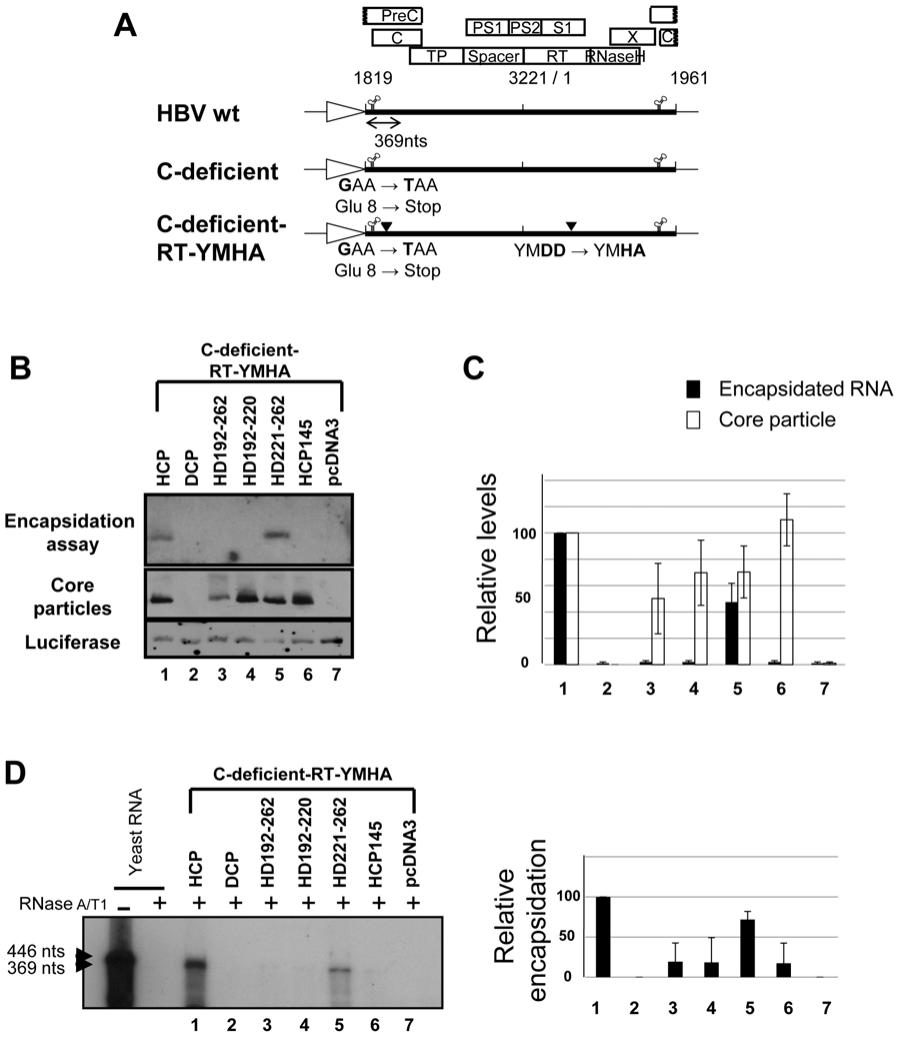
HBV **pgRNA encapsidation in core particles with chimeric C protein variants.** (A) Schematic diagram of HBV *wt* [27], C-deficient mutant, and C-deficient-RT-YMHA mutant. The C-deficient-RT-YMHA mutant is RT- and C-protein deficient due to mutation of the YMDD motif to YMHA, in addition to the presence of a stop codon at Glu 8 in the C ORF. The positions of point mutations are indicated as closed arrowheads. Four ORFs of HBV are shown at the top as open boxes. The CMV promoter is denoted by an open arrow. (B) Encapsidation assay to detect HBV nucleic acid *in situ* from disrupted core particles. To examine encapsidation by chimeric C protein variants, the C-deficient-RT-YMHA mutant was co-transfected into HuH7 cells with the pHCP, pDCP, pHD192–262, pHD192–220, pHD221–262, or pHCP145. HBV *wt* C protein from pHCP served as a positive control. Isolated core particles were electrophoresed through a 1% native agarose gel and transferred to nylon membrane. A ^32^P-labeled HBV DNA probe was hybridized to HBV nucleic acids in core particles after disruption of the particles *in situ*. Core particles were also detected as described for Figure 1B. (C) Relative levels of RNA encapsidation and core particle assembly by chimeric C protein variants. Relative levels of encapsidated RNA and core particles were measured with the Fujifilm Image Gauge V4.0 program. Relative levels of encapsidated RNA and core particles were compared with normalized transfection efficiencies (n = 3). (D) RNase protection assay (RPA) to detect encapsidated pgRNA. *In vitro* transcribed radiolabeled antisense RNA probe (446 nt) was hybridized overnight at 50°C with pgRNA from isolated core particles. Following RNase digestion, the protected pgRNA (369 nt), nt 1819–2187 of the HBV sequence, was run on a 5% polyacrylamide–8 M urea gel and visualized by autoradiography. Relative levels of encapsidated pgRNA were measured with the Fujifilm Image Gauge V4.0 program. Transfection experiments were repeated three times. The Renilla luciferase expression plasmid phRL-CMV was used as a transfection control and pcDNA3.1 was used to equalize the total amount of DNA transfected. The data represent the mean ± SD from three independent experiments.

Carboxyl-terminal amino acid sequences of HBV and DHBV C proteins exhibited 40% identity or 45% homology (Figure 1A), while full-length C protein sequences of HBV and DHBV were approximately 27% homologous. DHBV C protein contains an additional 29 amino acids that are absent in HBV C protein (Figure 1A). To investigate residues in the carboxyl-terminal nucleic acid binding domain of HBV C protein required for HBV replication, various chimeric C proteins were constructed by substituting the corresponding regions of DHBV C protein for the carboxyl-terminus of HBV C protein (Figure 1A). As positive and negative controls, the HBV and DHBV C protein expression plasmids pHCP and pDCP were first constructed and used as template for chimeric C protein construction. The HD221–262 C protein chimera was designed to substitute the carboxyl-terminal region from residues 221–262 of the DHBV C protein for the corresponding region from residues 146–185 of the HBV C protein, while the amino-terminal 145 amino acids of the HBV C protein were unchanged. The HD192–262 C protein chimera contains the amino-terminal 145 amino acids of HBV C protein and the carboxyl-terminus of DHBV C from residues 192–262 to include an additional 29 amino acids. The HD192–220 C protein variant has the entire HBV C protein sequence but an additional 29 amino acids which are part of DHBV C flexible linker region are inserted between residues 145–146 of the HBV C protein. The HCP145 construct was generated as a positive control for core particle assembly, but as a negative control for pgRNA encapsidation. HCP145–R127Q, the assembly-deficient variant, was constructed as a negative control for core particle assembly [23]. Construct transcription was controlled by the cytomegalovirus immediate early (CMV IE) promoter, and nuclear export of RNAs facilitated by the HBV PRE sequence [24] (Figure 1A). The C-deficient mutant that does not express C protein by the introduced stop codon (**T**AA) at Glu8 (GAA) (Figure 2A) and pHCP were used as control and/or reference. The Renilla luciferase expression plasmid phRL-CMV was co-transfected into HuH7 cells as a transfection control.

Following transfection of the C protein variants or C-deficient mutant indicated into HuH7 cells, C proteins from HBV *wt* and chimeric, mutated, and/or truncated variant constructs migrated as expected after SDS-PAGE and Western blotting with polyclonal anti-HBc antibody, but not the C-deficient mutant, as expected (Figure 1B, top panel). To exclude the possibility that the existence of HBV components such as pgRNA and P protein could affect assembly and/or stability of core particles, we transfected C protein variants alone, without the pgRNA expressing construct, into HuH7 cells. Most C protein chimeras were expressed similarly to or, occasionally, at higher levels than the HBV *wt* C protein from pHCP, except the C protein chimera from HD192–262 (Figure 1B, top panel, lane 3). Native agarose gel electrophoresis followed by Western blotting with polyclonal anti-HBc antibody revealed that core particles formed by chimeric C variants produced slightly different migration patterns (Figures 1B and 2B, second panel, lanes 3–6), suggesting that carboxyl-terminal nucleic acid binding domain sequence might affect core particle formation to some extent, even though the amino-terminal assembly domain remained intact in these chimeric C variants. DHBV C protein and core particles could not be detected with anti-HBc antibody (Figure 1B and C, lane 2). Also, the assembly-deficient mutant HCP145–R127Q could not form core particles [23], even though HCP145–R127Q C protein was compatible with HCP145 C protein (Figure 1B and C, lanes 6 and 7). When levels of core particle formation were compared with C protein expression by normalization to the phRL-CMV transfection control, all variants exhibited similar patterns except the assembly-deficient mutant (Figure 1C). The very inefficient core particle formation by HD192–262 may have been due to poor C protein expression (Figure 1B and C, lane 3). Furthermore, the migration pattern displayed by core particles formed with the HD192–262 C chimera was slightly slower than those of other core particles (Figures 1B, 2B, 5A second panels, and 6 bottom panel), suggesting that HD192–262 core particles may be less stable [25]. Alternatively, it might be caused by the differences in net charges [26].

### HBV RNA Encapsidation in Core Particles with C Protein Chimeras

To examine RNA encapsidation by assembly-competent chimeric C variants, various C protein chimeras were co-transfected into HuH7 cells with the C-deficient-RT-YMHA mutant (Figure 2A). To ensure that the nucleic acids within core particles hybridized *in situ* are encapsidated RNA, not synthesized HBV DNA, the C-deficient-RT-YMHA mutant was used for co-transfection experiments. The conserved YMDD reverse transcriptase motif was modified to YMHA [27] in the C-deficient mutant background in the C-deficient-RT-YMHA mutant; thus, C protein deficient and RT reaction-deficient. In this system, C proteins were supplied in *trans* from C protein chimeras to trans-complement C-deficient-RT-YMHA mutant, and pgRNA and HBV P protein for pgRNA encapsidation were provided from C-deficient-RT-YMHA mutant to trans-complement C protein chimera. HCP and C-deficient-RT-YMHA co-transfected cells were used as a positive control that complements one another. Core particles from co-transfected cells (Figures 2B, second panel, and 2C) were assembled with efficiency and stability similar to those from singly transfected cells (Figures 1B and C), indicating that core particle stability might not be affected by the existence of pgRNA and P protein. We designated HCP145 as the encapsidation-negative control based on previous reports [3], [28]. Encapsidated RNAs were detected only from HD221–262 C variant and C-deficient-RT-YMHA co-transfected cells (Figures 2B, top panel, and 2C; see lane 5 in each). Encapsidated RNAs were not detected from HD192–262 and HD192–220 C variant co-transfected cells (Figures 2B, top panel, and 2C).

RNase Protection assay (RPA) was also performed with 5′-end specific probe to show encapsidation of HBV RNA by chimeric C variants. Consistent with encapsidation assays (Figure. 2B, top panel), encapsidation of HBV RNA was only detected in HD221–262 C variant and C-deficient-RT-YMHA co-transfected cells (Figure 2D, lane 5). RPA and encapsidation assay results indicated that 40% identity or 45% homology in the carboxyl-terminus of HBV C protein was sufficient for pgRNA encapsidation.

### HBV DNA is Synthesized in Core Particles by the HD221–262 C Variant

**Figure 3:**
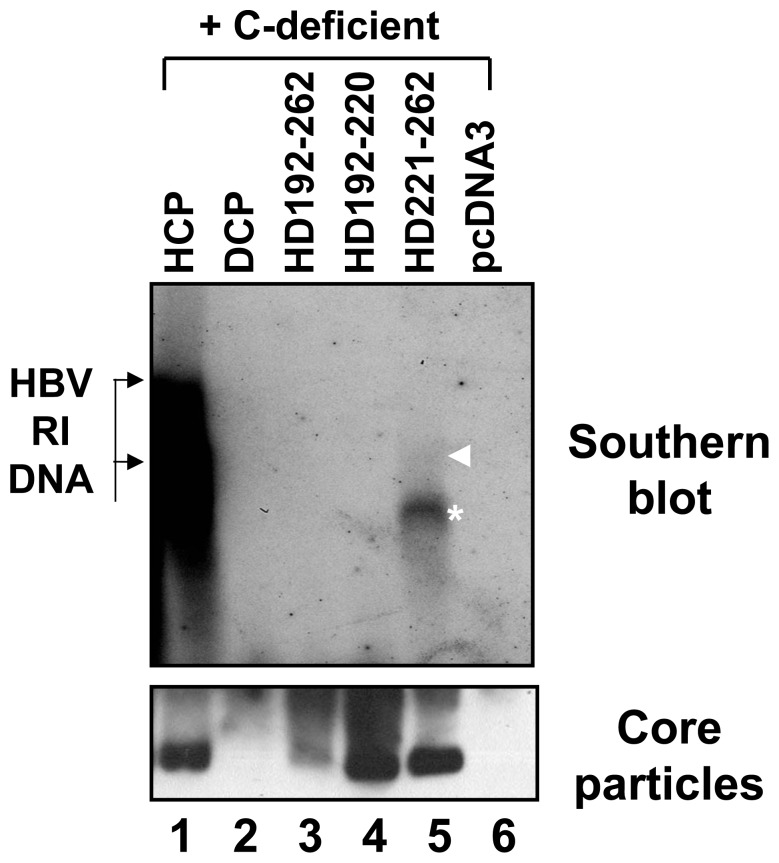
HBV DNA synthesis in core particles with chimeric C protein variants. To examine HBV DNA synthesis in core particles with chimeric C variants, the C-deficient mutant and the pHCP, pDCP, pHD192–262, pHD192–220, or pHD221–262 were co-transfected into HuH7 cells. HBV DNA was extracted from isolated core particles and Southern blot analysis performed. Briefly, HBV DNA was separated, transferred to nylon membranes, hybridized with a random-primed ^32^P-labeled HBV specific probe, and subjected to autoradiography. Transfection experiments were repeated more than three times. The HBV replicative intermediate (RI) DNA is marked. Core particle formation (bottom panel) was determined as described for Figure 1B.

To further investigate whether this chimeric C protein could support HBV DNA synthesis, Southern blot analysis was performed. As expected, HBV DNA was detected only from HuH7 cells co-transfected with the HD221–262 chimera and C-deficient mutant, and migrated faster than major replicative intermediate (RI) HBV DNAs (Figure 3, lane 5, asterisk), which includes RC, double-stranded linear (DL), and single-stranded (SS) DNA. A shorter exposure to visualize HBV full-length RI DNA from HCP and C-deficient mutant co-transfected cells clearly revealed production of smaller DNA species from HuH7 cells co-transfected with the HD221–262 chimera and C-deficient mutant (data not shown). A faint band, potentially corresponding to one of major RI DNA, was also detected by longer exposure (Figure 3, lane 5, arrowhead). However, full-length RC HBV DNA was not detected (Figure 3). This result demonstrated that 40% identity or 45% homology at the carboxyl-terminus of the C protein was sufficient to support HBV DNA synthesis, but not that of full-length HBV DNA.

### Core Particle Formation and RNA Encapsidation by Additional Chimeric C Variants

**Figure 4:**
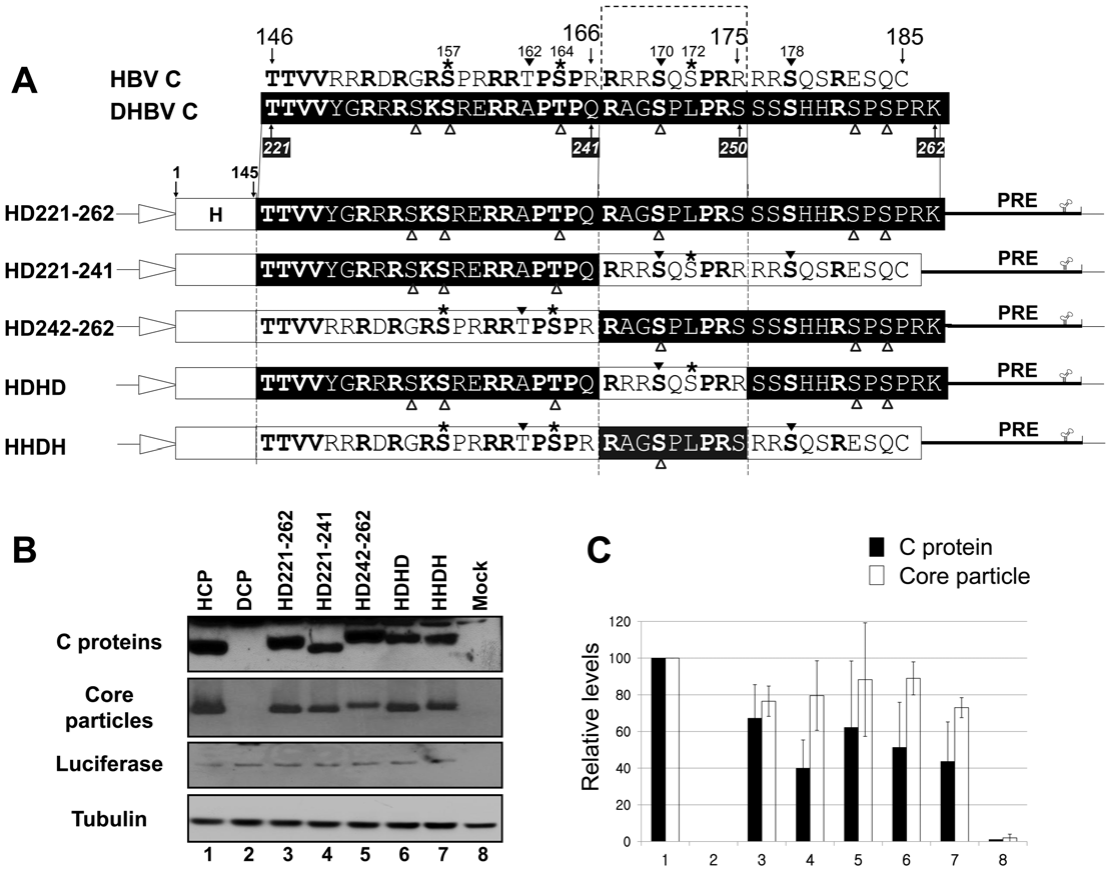
Expression and core particle assembly of additional C protein variants. (A) Aligned amino acid sequences of carboxyl-terminal domains of HBV and DHBV C proteins and schematic diagrams of additional chimeric C protein variant constructs. Amino acids in bold are identical or homologous. SRPK and PKA phosphorylation sites in the HBV genome are marked with asterisks and arrowheads, respectively. DHBV phosphorylation sites [38], [41] are marked with open arrowheads. The amino acid sequences of HBV and DHBV C protein are presented as open and closed boxes, respectively. The cytomegalovirus immediate early (CMV IE) promoter is represented as an open arrow. PRE, post-transcriptional regulatory element. (B) Expression of chimeric C proteins and core particle assembly by additional chimeric C protein variants. To examine C protein expression by HBV variants with chimeric C sequence, Western blotting was performed on lysates from HuH7 cells and HuH7 cells transfected with pHCP, pDCP, pHD221–262, pHD221–241, pHD242–262, pHDHD, or pHHDH variants, as described for Figure 1B (top panel). Core particle formation by C protein variants was detected as described for Figure 1B (second panel). Transfection experiments were repeated four times. As the respective transfection and loading controls, luciferase (third panel) and α-tubulin (bottom panel) levels were determined as described for Figure 1B. (C) Relative levels of C protein expression and core particle assembly by additional chimeric C protein variants. Relative levels of C proteins, core particles, and luciferase were measured with the Fujifilm Image Gauge V4.0 program. Relative levels of C protein variant expression and core particle assembly were compared with normalized transfection efficiencies. The data represent the mean ± SD from four independent experiments.

The detection of fast-migrating, smaller than full-length RI HBV DNA, as a major DNA species from HD221–262 co-transfected cells led us to attempt identification of the motif critical for full-length HBV DNA synthesis. To accomplish this, we constructed and analyzed new chimeric C variants with varying lengths of the DHBV C protein carboxyl-terminus. Since it has been reported that a carboxyl-terminal deleted C164 variant (C166 in adw) can support pgRNA encapsidation and DNA synthesis, even though it is predominantly spliced [6], [9], we constructed the HD221–241 C variant, with residues 221–241 of DHBV C substituted for residues 146–166 of HBV C, as well as the HD242–262 C variant, with residues 242–262 of DHBV C in place of residues 167–185 of HBV C (Figure 4A). Also, prompted by the suggestion that residues 165–173 (167–175 in *adw*) of the HBV C protein were important for selective and/or productive viral RNA encapsidation, we further constructed the HDHD C chimeric variant with residues 221–241 and 251–262 of DHBV C in the position of residues 146–166 and 176–185 of HBV C, respectively, and the reciprocal HHDH chimeric C variant with residues 242–250 of DHBV C in the position of residues 167–175 of HBV C (Figure 4A). Expression of chimeric C proteins and assembly of core particles were analyzed from C variants transfected HuH7 cells (Figures 4B, top and second panels, and 4C). Variation was evident, however, all C protein variants were expressed and core particles assembled (Figure 4B and C). Similar to the core particles of the HD192–262 C variant, the core particles of the HD242–262 and HHDH C variants seemed to migrate slightly more slowly than core particles of pHCP C protein (Figure 4B, second panel, lanes 5 and 7).

**Figure 5:**
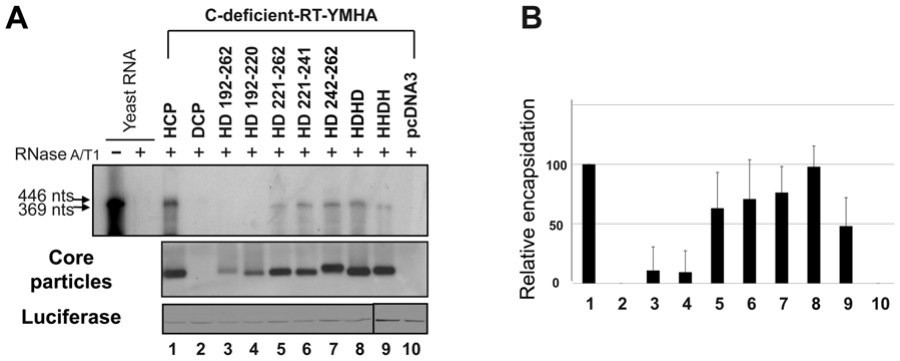
pgRNA encapsidation in core particles by additional C protein variants. (A) RPA to detect encapsidated pgRNA. To detect the pgRNA encapsidated by chimeric C protein variants, the C-deficient-RT-YMHA mutant and the C protein chimeras pHCP, pDCP, pHD192–262, pHD192–220, pHD221–262, pHD221–241, pHD242–262, pHDHD, or pHHDH were co-transfected into HuH7 cells. RPA (top panel) was performed as described for Figure 2D. Core particle formation (second panel) and luciferase levels (bottom panel) were determined as described for Figure 1B. Transfection experiments were repeated three times. (B) Relative levels of encapsidated pgRNA and core particle assembly by additional chimeric C protein variants. Relative levels of encapsidated pgRNA, core particles, and luciferase were measured with the Fujifilm Image Gauge V4.0 program. Relative levels of encapsidated pgRNA and core particle assembly were compared to normalized transfection efficiencies. The data represent the mean ± SD from three independent experiments.

To examine RNA encapsidation by these C variants, RPAs (Figure 5) and encapsidation assays (data not shown) were also performed in chimeric C variants and C-deficient-RT-YMHA mutant co-transfected HuH7 cells. Core particles from these co-transfections exhibited similar assembly efficiency and migration patterns as those of singly transfected cells (see Figure 4B; Figure 5A, second panel). We could not detect significantly increased RNA encapsidation from cells co-transfected with these additional C variants compared to that from HD221–262 co-transfected cells (Figure 5A and B). pgRNA from cells co-transfected with the HDHD C variant and C-deficient-RT-YMHA mutant displayed slightly increased RNA encapsidation (Figure 5, lane 8). Since RPA with a 5**′**-end specific probe could not distinguish spliced encapsidated RNA from unspliced full-length pgRNA, encapsidation efficiency determined by RPA represented total encapsidated HBV RNA rather than full-length pgRNA incorporated into core particles. Consistent with RPA, RNAs encapsidated within core particles *in situ* were also detected from cells co-transfected with HD221–262, HD221–241, HD242–262, HDHD, or HHDH C variant and the C-deficient-RT-YMHA mutant (data not shown).

To our surprise, pgRNA encapsidation from HHDH C variant and C-deficient-RT-YMHA mutant co-transfections was less efficient than from HD221–262 C variant and C-deficient-RT-YMHA mutant co-transfections (Figure 5, and data not shown). The HHDH C variant contains most HBV C protein sequences, with the substitution only of a nine amino-acid motif from residues 242–250 (^242^R***AG***S***PL***PR***S*** ^250^) of DHBV C for residues 167–175 (^167^R***RR***S***QS***PR***R*** ^175^) of HBV C. A closer inspection of the HHDH C variant revealed that only five amino acids (in italics and underlined) differed from the HBV C protein sequence. These results indicated that amino acid residues 167–175 of the HBV C protein were critical for efficient pgRNA encapsidation, and amino acid residues 150–166 and 176–185 of HBV C protein were not essential, as long as the 40% amino-acid identity or 45% homology or the several critical residues, presumably within the conserved region, were maintained.

### Full-length HBV DNA is Synthesized in Core Particles by the HDHD C Variants

**Figure 6 pone-0041087-g006:**
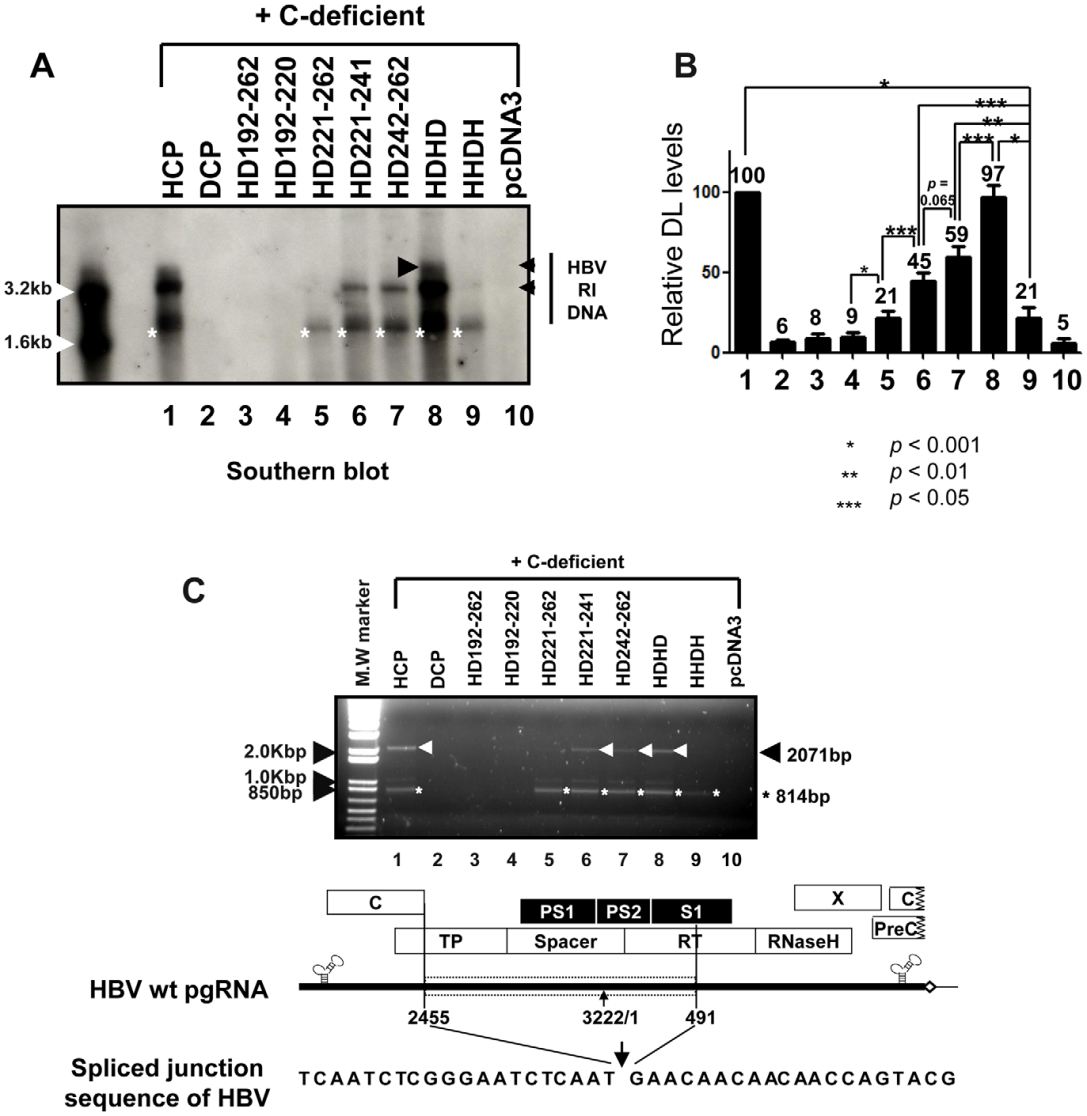
Full-length HBV DNA synthesis in core particles with additional chimeric C protein variants. (A) To examine synthesis of HBV DNA in core particles with chimeric C variants, the C-deficient mutant and the C protein chimeras pHCP, pDCP, pHD192–262, pHD192–220, pHD221–262, pHD221–241, pHD242–262, pHDHD, or pHHDH were co-transfected into HuH7 cells. HBV DNA was extracted from isolated core particles and Southern blot analysis performed as described for Figure 3. Transfection experiments were repeated five times. The HBV replicative intermediate (RI) DNA is marked. Core particle formation (bottom panel) was determined as described for Figure 1B. (B) Relative levels of HBV double-stranded linear (DL) DNA from isolated core particles were measured with the Fujifilm Image Gauge V4.0 program and compared after normalization to transfection efficiencies (top right panel). The data represent the mean ± SD from five independent experiments. * p<0.001, ** p<0.01, *** p<0.05 (n = 5). (C) PCR and sequence alignment of the spliced junction of DNAs from isolated core particles. HBV DNA was extracted from isolated core particles and PCR was performed. The 814 base-pair (bp) DNA that was 1257 nt smaller than 2,071 bp of full-length HBV DNA and full-length HBV DNA were amplified (arrowheads).

**Figure 7:**
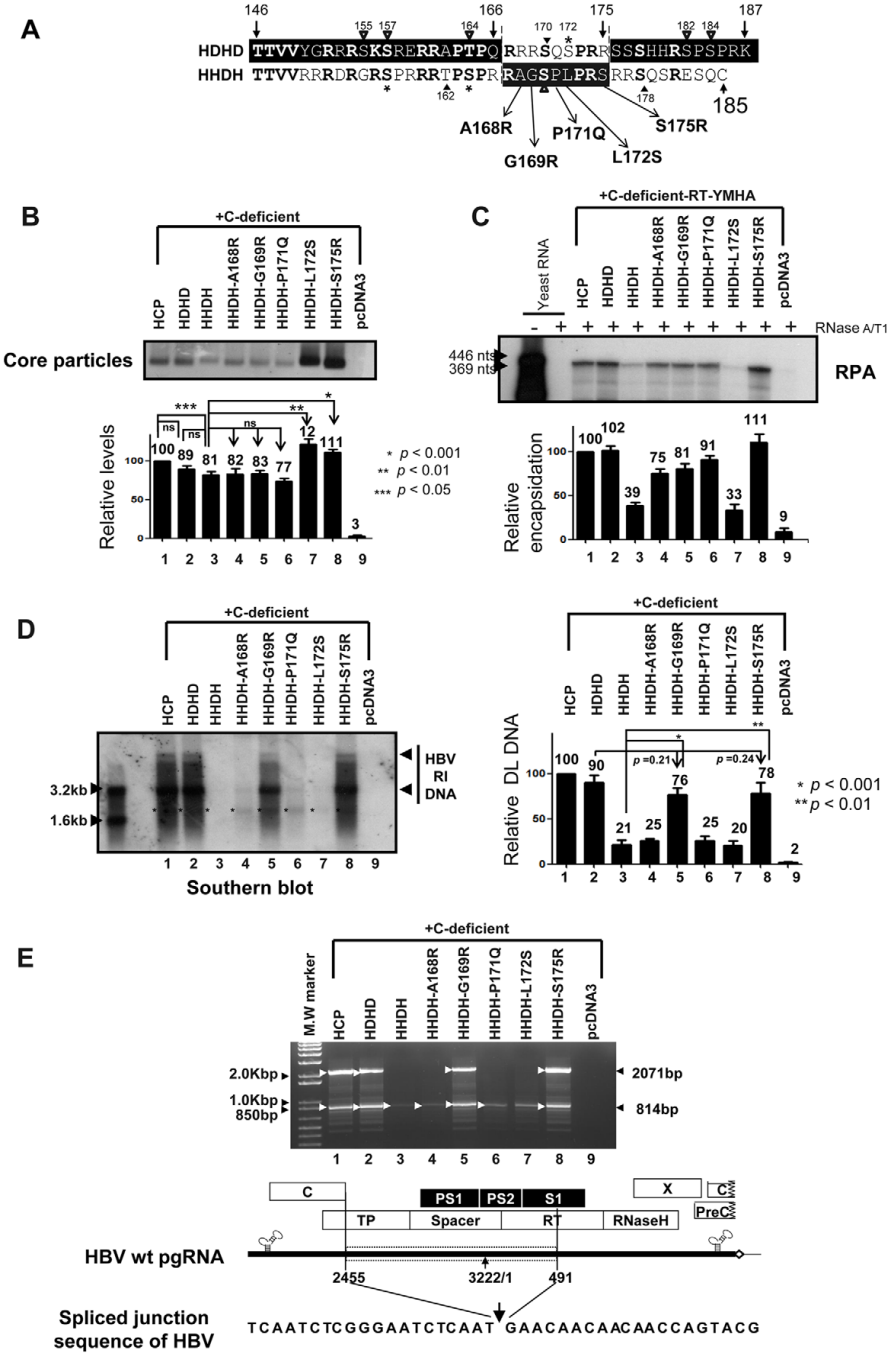
HBV core particle formation, pgRNA encapsidation, and HBV DNA synthesis by C variants. (A) Aligned amino acid sequences of HDHD and HHDH and amino acid substitutions in the HHDH-derived C variants HHDH-A168R, HHDH-G169R, HHDH-P170Q, HHDH-L172S, and HHDH-S175R. Amino acids in bold are identical or homologous. SRPK and PKA phosphorylation sites of HBV are marked with asterisks and arrowheads, respectively. Phosphorylation sites of DHBV [38], [41] are marked with open arrowheads. Amino acid sequences of HBV and DHBV C proteins are presented as black and white letters, respectively, on contrasting background. (B-D) HBV core particle formation, pgRNA encapsidation, and HBV DNA synthesis by C variants. To examine HBV core particle formation (B), pgRNA encapsidation (C), and HBV DNA synthesis (D) in core particles with the C-deficient or C-deficient-RT-YMHA mutants and pHCP or the C protein chimeras, pHDHD, pHHDH, HHDH-A168R, HHDH-G169R, HHDH-P170Q, HHDH-L172S, or HHDH-S175R, were co-transfected into HuH7 cells. pcDNA3.1 was used to equalize the amount of DNA transfected. (B) Core particle formation and luciferase levels (data not shown) were determined as described for Figure 1B. The data represent the mean ± SD (n = 5). * p<0.001, ** p<0.01, and * p<0.05 (n = 5). (C) To examine pgRNA encapsidation, RPA was performed as described for Figure 2D. The data represent the mean ± SD from four independent experiments. (D) HBV DNA was extracted from isolated core particles and Southern blot analysis performed as described for Figure 3. The HBV replicative intermediate (RI) DNA is marked. DNAs from spliced RNAs are indicated by asterisks. Relative levels of core particles and encapsidated pgRNA and HBV DL DNA from isolated core particles were measured with the Fujifilm Image Gauge V4.0 program and compared after normalization to transfection efficiencies. The data represent the mean ± SD from five independent experiments. * p<0.001 HHDH *vs* HHDH-G169R, ** p<0.01 HHDH *vs* HHDH-S175R, *p* = 0.21 HDHD *vs* HHDH-G169R, or *p* = 0.24 HDHD *vs* HHDH-S175R (n = 5). (E) PCR and sequence alignment of the spliced junction. HBV DNA was extracted from isolated core particles and PCR was performed as described for Figure 6C. The 814 base-pair (bp) DNA that was 1257 nt smaller than 2,071 bp of full-length HBV DNA and full-length HBV DNA were amplified (arrowheads).

To identify the motif required for full-length HBV DNA synthesis, we analyzed DNA from HuH7 cells co-transfected with these additional chimeric C variants and C-deficient mutant. We clearly detected full-length DL DNA in HD221–241, HD242–262, and HDHD co-transfected HuH7 cells (Figures 6A and 6B, lanes 6–8). RC DNA was detected only from HDHD C variant co-transfected HuH7 cells (Figure 6A, lane 8). Full-length DNA was barely detectable from HHDH co-transfected cells. Collectively, these results further suggest that the amino acid residues 167–175 of the HBV C protein (^167^RRRSQSPRR^175^) are important for full-length DNA synthesis, while residues 150–166 and 176–185 are not. Since the HHDH C variant encapsidated pgRNA less efficiently (Figures 5 and 7C), the low level of DNA produced by cells co-transfected with the HHDH C variant and C-deficient mutant likely reflects this low encapsidation efficiency (Figures 6A, 6B, and 7D).

Similar to co-transfection with HD221–262 (Figure 3), we also detected small-sized DNA from all cells co-transfected with C variants (Figure 6A, asterisks). We speculate that these small-sized DNAs were synthesized from spliced RNA, since HBV DNAs produced by the carboxyl-terminally deleted C164 variant are predominantly from spliced RNA [6], [9]. When we used a probe corresponding to the regions most frequently removed during splicing [29], [30], the intensities of these small DNA forms were significantly decreased in C variant co-transfected cells (data not shown) and were barely detectable in core particles from HD221–262 and HHDH co-transfected cells (data not shown); this is consistent with the forms synthesized by HD221–241, HD242–262, and HDHD variant C proteins primarily comprising DNA from spliced RNA. We also used a minus-strand RNA probe to detect plus-stranded HBV DNA (data not shown) and a plus-strand RNA probe to detect minus-stranded HBV DNA (data not shown). Both probes detected small-sized DNA, indicating that small DNA was double-stranded DNA synthesized from spliced RNA (data not shown). To further analyze small-sized DNA in detail, (Figure 6A, asterisks), polymerase chain reaction (PCR) was performed from core particles isolated from C-deficient mutant and various C variants co-transfected HuH7 cells. Consistent with the results from Southern blotting using strand-specific probes (data not shown) and spliced-out region specific probe (data not shown), small-sized DNA was amplified from HCP, HD221–262, HD221–241, HD242–262, HDHD, HHDH C variants co-transfected cells (Figure 6C, lanes 1, 5–9), further indicating that small DNA was from spliced RNA. Consistent with Southern blotting (Figure 6A), full-length DNA was also amplified from HCP, HD221–241, HD242–262, and HDHD C variants co-transfected cells (Figure 6C, lanes 1, 6–8), but not or hardly detectable from DCP, HD192–262, HD192–220, HD221–262, HHDH C variant co-transfected cells (Figure 6C, lanes 2–4, and 9). Sequence analysis revealed that the PCR-amplified small DNA was spliced from nucleotides (nt) 2455–491, one of the most spliced sites, deleting 1257 nt (Figure 6C) [30]. Since the intensity of the small-sized DNA from cells co-transfected with HHDH was even weaker than those from cells co-transfected with HD221–241 and HD242–262 (Figure 6A and C, lanes 6 and 7 *vs* 9), we further speculate that the HHDH may be less competent to encapsidate spliced RNA also (Figure S2).

### Residues R169 and R175 are Important for HBV Replication

To identify the amino acid residues from 167–175 of HBV C protein (^167^R***RR***S***QS***PR***R*** ^175^) that are important for rescue of full-length HBV DNA synthesis from HHDH C variant co-transfected cells, we singly altered amino acids in the HHDH background motif, comprising ^167^R***AG***S***PL***PR***S*** ^175^, to the corresponding residues in HBV, resulting in HHDH-A168R, HHDH-G169R, HHDH-P171Q, HHDH-L172S, and HHDH-S175R C variants (Figure 7A). Core particle formation was examined by particle Western blotting from HuH7 cells co-transfected with HHDH-A168R, HHDH-G169R, HHDH-P171Q, HHDH-L172S, or HHDH-S175R C variants and the C-deficient mutant (Figure 7B). Core particle assembly efficiency of the HHDH-A168R, HHDH-G169R, and HHDH-171Q was not restored to the level of HBV *wt* C protein and, although not significant, was slightly less efficient than that of the HDHD C variant (Figure 7B, n = 5). However, core particle assembly efficiency of the HHDH-L172S (*p*<0.001, n = 5) and HHDH-S175R (*p*<0.0001, n = 5) was restored and was more efficient than that of the HDHD C variant and HBV *wt* C protein (Figure 7B).

RNA encapsidation was examined by RPA in HuH7 cells co-transfected with additional C variants and the C-deficient-RT-YMHA mutant (Figure 7C, n = 4). Consistent with Figure 5, RNA encapsidation by the HHDH C variant was markedly reduced compared to HBV *wt* C protein or HDHD C variants (Figure 7C, lanes 1 and 2 *vs* lane 3). HHDH-A168R, HHDH-G169R, and HHDH-P171Q C variants could rescue pgRNA encapsidation, although less efficiently than HBV *wt* C protein or the HDHD C variant (Figure 7C). The HHDH-S175R could encapsidate pgRNA more efficiently than HBV *wt* C protein (Figure 7C, lanes 1 *vs* 8). However, the HHDH-L172S encapsidated pgRNA very poorly (Figure 7C, lane 7), even though core particles by HHDH-L172S were assembled more efficiently than HBV *wt* C protein or the HDHD C variant (Figure 7B and C, lanes 7).

HBV DNA synthesis was also examined by Southern blotting, using a probe specific for full-length HBV, of cells co-transfected with HHDH-A168R, HHDH-G169R, HHDH-P171Q, HHDH-L172S, or HHDH-S175R C variants and the C-deficient mutant (Figure 7D). Full-length RC HBV DNA synthesis was observed from cells co-transfected with HCP, HDHD, HHDH-G169R, or HHDH-S175R C variants and the C-deficient mutant, indicating the importance of the R169 and R175 residues for full-length RC HBV DNA synthesis (Figure 7D, lanes 1, 2, 5, and 8). However, HBV DNA synthesis was not rescued by HHDH-A168R, HHDH-P171Q, or HHDH-L172S C variants (Figure 7D, lanes 4, 6, and 7). The low level of HBV DNA synthesis by the HHDH-L172S C variant was due to its very inefficient pgRNA encapsidation (Figures 7C and D, lanes 7). Since residues R167, S170, P173, and R174 are identical to the corresponding regions from DHBV (^242^R***AG***S***PL***PR***S*** ^250^ in DHBV vs ^167^R***RR***S***QS***PR***R*** ^175^ in HBV), their importance remains to be determined.

Polymerase chain reaction (PCR) was also performed as in Figure 6C to further examine the small-sized DNA (see Figure 7D, asterisks). Consistent with Figure 6C and the result from spliced-out region specific probe (data not shown), small-sized DNA was amplified from C variants co-transfected cells (Figure 7E, lanes 1–8), further indicating that small-sized DNA was from spliced RNA. In consistent with Southern blotting (Figure 7D), full-length DNA was also amplified from HCP, HDHD, HHDH-G169R, and HHDH-S175R co-transfected cells (Figure 7E, lanes 1, 2, 5, and 8), but not from HHDH, HHDH-A168R, HHDH-P171Q, or HHDH-L172S co-transfected cells (Figure 7E, lanes 3, 4, 6, and 7). Consistent with the result from Figure 6C, sequence analysis revealed that one of the most spliced sites, nucleotides (nt) 2455–491, was deleted by splicing (Figure 7E) [30].

To further confirm this result, RPA was also performed to discriminate encapsidated full-length pgRNA from spliced RNA using a spliced-out region probe which encompasses nt 2689–3092 of HBV sequence [29] (Figure S2). If chimeric C variants encapsidated spliced RNA more efficiently than full-length pgRNA, the encapsidation level by spliced-out region probe would be low compare to that by 5**′**-end specific probe. As shown in Figure S2, the encapsidation efficiencies did not differ significantly between spliced-out region and 5**′**-end specific probes (Figure 7C *vs* Figure S2), we could conclude that the low level of HBV DNA synthesis by the HHDH and HHDH-L172S C variant was due to very inefficient pgRNA encapsidation (Figures 7C-D and Figure S2, lanes 3 and 7).

## Discussion

In this study, chimeric C variants by substituting the carboxyl-terminal regions of HBV C protein with the corresponding regions of DHBV C protein were generated and core particle assembly, pgRNA encapsidation, and HBV DNA synthesis were examined. Unlike DHBV C protein, which fails to *trans*-complement HBV C protein [20], various chimeric C variants could *trans*-complement HBV replication, including the HD221–262 with carboxyl-terminal 42 amino acids of DHBV C protein for those in HBV. We therefore hypothesize that the amino-terminus of HBV C protein may interact with viral or host components to form a tertiary structure and/or to support HBV replication.

### Core Particle Assembly for HBV Replication

All chimeric C variants can assemble into core particles as long as the N-terminal 145 residues of HBV C proteins are intact (Figure 1B). However, core particles formed by chimeric C variants migrated slowly and demonstrated differing assembly competencies (Figures 1B, 2B, 4B, 5A, and 6A), suggesting that the carboxyl-terminal nucleic acid binding domain may affect core particle formation to some extent or interact with the amino-terminal assembly domain for particle stability. The presence of slowly migrating core particles (HD192–262, HD242–262, and HHDH C chimeras) suggests that these core particles might be less stable or differ in net charge, thus affecting HBV replication [9], [25], [27]. HHDH core particles migrated more slowly than HDHD, providing evidence of inefficient pgRNA encapsidation and DNA synthesis (Figures 5–7). However, this speculation did not apply to all C chimeras, since pgRNA encapsidation and HBV DNA synthesis by HD221–242 and HD242–262 were similar, even though core particle migration patterns were different (Figures 5 and 6).

Core particles assembled from truncated C proteins or those with insertions are unstable [6], [9], [31]; destabilization through insertional mutagenesis may explain the failure of *trans*-complementation by HD192–262 and HD192–220. It is still possible to speculate that the insertion of 29 residues in HD192–220 destabilizes core particles, thus preventing protection of encapsidated pgRNA from nuclease treatment during core particle isolation. Since HD192–262 has more extensive alterations, these alterations may cause the destabilizations of chimeric C protein and/or core particles or reduce the efficiency of core particle assembly.

### Sequence Conservation in the Carboxyl-terminal Domain of Hepadnavirus C Protein

Using a series of C protein carboxyl-terminal deletion mutants, Le Pogam et al. [9] suggested that residues 167–175 (165–173 of *ayw*) of HBV C protein are important for selective and/or productive viral RNA encapsidation by charge balance and core particle stability through the arginine-rich domain. The carboxyl-terminal 10 amino acids of HBV C protein are dispensable for HBV DNA replication [6], [9]. The present study extends these results by showing that residues 167–175 (165–173 of *ayw*), as well as the 62% homologous residues from 146–166, of HBV C protein are sufficient for full-length HBV DNA synthesis using the HDHD C variant (Figures 6A and 6B). From the 27% homology between HBV and DHBV C proteins, the carboxyl-terminus is 45% homologous or 40% identical, and residues 146–166 are 62% homologous, suggesting that several critical residues from 146–166 may be conserved or have coevolved to encapsidate pgRNA and subsequently synthesize DNA.

Even though our results also demonstrated that residues 167–175 (165–173 of *ayw*) of HBV C protein are important for HBV replication, several questions are still unanswered. First, if these residues are solely essential for HBV replication as long as residues 146–166 are at least 62% homologous, the replication efficiencies of HD221–241 and HDHD should be similar, and the former should engage in full-length RC DNA synthesis, as does the HDHD. Second, replication of the HD242–262 should be inefficient, similar to that of the HD221–262 and/or HHDH. However, HD221–241 and HD242–262 exhibited similar replication efficiencies; replication efficiency was improved relative to HD221–262. Although not significant (*p* = 0.065, n = 5), HBV DNA synthesis was little more efficient by HD242–262 than HD221–241 (Figure 6B).

### Putative Phosphorylation Sites in the Carboxyl-terminal Domain of HBV C Protein

Hepadnavirus C proteins are heavily phosphorylated [32], [33]–[36]. The C protein of DHBV is phosphorylated at six sites (S230, S232, T239, S245, S257, and S259) on an S/TP motif within the carboxyl-terminal domain [37], [38]. Three phosphorylation sites (S155, S162, and S170 in subtype *ayw* and S157, S164, and S172 in subtype *adw*) in the carboxyl-terminal domain of HBV C protein have been identified as having an SPRRR motif [34]. Several intracellular protein kinases such as protein kinase C [39], the cyclin-dependent kinase cdc2 [40], the 46 kDa serine protein kinase [41], and serine/arginine protein-specific kinases 1 (95 kDa SRPK1) and 2 (105 kDa SRPK2) [42] have been shown to phosphorylate these serine residues *in vitro*. The synthesis of smaller than full-length DNA was also demonstrated for major phosphorylation-site mutants (S155E, S162E, and S170E) [6]. Phosphorylation at these sites is important for pgRNA encapsidation and HBV replication [6], [32], [33], [43] and these serine phospho-acceptor sites contribute pleiotropically toward modulating HBV replication [8]. Three additional putative cAMP-dependent protein kinase A (PKA) phosphorylation sites (RRXS/T: T162, S170, S178) have been identified, and two α-type CK2-activated PKAs (PKAIα and PKAIIα) phosphorylate both S170 and S178 *in vitro* in the absence of cAMP [44].

Phosphorylation and dephosphorylation states may not be drastically altered in the C protein chimeras, since both HBV and DHBV C proteins have six phospho-acceptor sites (Figures 1A and 4A). However, for the HHDH, the S172L and R175S substitutions may have reduced the number of putative phosphorylation sites to four (Figure 4A). Also, even though S178 was retained in the HHDH, the ^175^ ***S***RRS^178^ could have disrupted ^175^RRRS^178^ motif, the putative PKA phosphorylation site at S178, contributing to inefficient pgRNA encapsidation and HBV replication (Figures 5, 6 and 7). HDHD, however, have seven putative phosphorylation sites, ensuing efficient pgRNA encapsidation and HBV replication.

### Arginine-rich Domains in the Carboxyl-terminus of HBV C Protein

The carboxyl-terminal domain of HBV C protein has 16 (ayw) or 17 (adw) arginine residues with four clusters (^150^RRR^152^, ^159^RRR^161^, ^166^RRRR^169^, and ^174^RRRR^177^) comprising arginine-rich domains (ARD) I–IV and conferring a net positive charge (Figure 4A). The carboxyl-terminal domain of DHBV C protein, in contrast, has 12 positively charged amino acids (arginine or lysine) but does not conserve the four ARDs, although the ^227^RRR^229^ and ^233^RERR^236^ motifs may be equivalent to ARD-I and -II (Figure 4A). Recently, mutagenesis of the ARDs of HBV C protein demonstrated their pleiotropic contribution to HBV replication [7]. R to A (RRRR→AAAA) mutation in ARD-III impaired in pgRNA encapsidation and minus-strand DNA template switching most strikingly [7].

Since ARD-I and -II remained intact in HHDH, inefficient pgRNA encapsidation and subsequent poor replication might be attributed to the ^166^RR***AG*** ^169^ (ARD-III) and ^174^R***S***RR^177^ (ARD-IV) changes. In HD242–262, ARD-III and ARD-IV were disrupted to ^166^RR***AG*** ^169^ and ^174^R***SSS*** ^177^, respectively, but HBV replication was more efficient than HHDH. HDHD has ^158^RERR^161^ (ARD-II), ^166^ ***Q***RRR^169^ (ARD-III), and ^174^RR***SS*** ^177^ (ARD-IV), indicating that these changes may be tolerated to maintain full-length DNA synthesis. Also, HD221–241, HD242–262, HHDH, and HDHD have 15, 14, 15, and 14 positively charged amino acids (arginine or lysine) respectively, in their carboxyl-terminal domains (Figure 4A).

### Important Amino Acids in the Carboxyl-terminal Domain of HBV C Protein for HBV Replication

The HHDH-A168R rescued pgRNA encapsidation, even though HBV DNA synthesis was not fully rescued (Figures 7C and 7D, lane 4), suggesting that R168 itself, partial restoration of ARD-III (^166^RR***R*** ^168^), and/or S170 in ^167^R***R***GS^170^ motif (a putative PKA phosphorylation site) may be important for encapsidation, but are not sufficient to support HBV DNA synthesis. HHDH-G169R restored pgRNA encapsidation and HBV DNA synthesis, suggesting that the ^167^RA***R***SPLPRS^175^ motif might be sufficient to form a replication-competent tertiary structure or protein-protein and/or protein-nucleic acid interactions (Figure 7). The HHDH-P171Q showed a similar phenotype to the HHDH-A168R, suggesting that Q171 itself may not be important for HBV DNA synthesis. The HHDH-L172S failed to rescue pgRNA encapsidation and HBV DNA synthesis, which is not consistent with previous reports, showing that S172 (S170 for *ayw*), the putative SRPK phosphorylation site, is important for HBV replication at various stages [6], [8], [32], [33], [43]. We hypothesize that S172 in ^172^ ***S***P^173^ may not be critical to enhance impaired HBV replication, or that ^170^SP^171^ compensates such that either ^172^ ***S***P^173^ is not necessary or the SP motif repeated in^ 170^SP***S***P^173^ has a negative effect. The HHDH-S175R restored pgRNA encapsidation and HBV DNA synthesis, suggesting that R175 itself, restoration of ARD-IV (^174^R***R***RR^177^), S178 phosphorylation site (comprising a putative PKA in the ^175^ ***R***RRS^178^ motif), or the positively charged ^174^R***R*** ^175^ [9] may be important. Alternatively, the ^167^RAGSPLPR***R*** ^175^ motif might be sufficient. To note, R169 and R175 are conserved in all HBV strains. Cumulatively, ARDs, phosphorylation sites, and ^167^RRRSQSPRR^175^ motif in the carboxyl-terminal domain of HBV C protein may interact to influence each other’s conformation, or that of the amino-terminus, to form a replication-competent tertiary structure or to support intra-molecular or inter-molecular protein interactions, protein-nucleic acid interactions, and/or interactions with host proteins promoting efficient HBV replication, with pleiotropic contributions at various stages of replication. We were unable to examine whether the R167, S170, P173, and R174 residues were critical for full-length RC DNA synthesis because the corresponding residues of DHBV (^242^ *R* **AG** *S* **PL** *PR* **S** ^250^) are identical. Their contribution will be the subject of future study.

## Materials and Methods

### HBV DNA Construction

The partially redundant *wt* HBV subtype *adw* R9 plasmid construct pPB was used as a template for generation of HBV DNA constructs using PCR-based mutagenesis [27]. An HBV *wt* C protein construct containing the HBV *wt* C open reading frame (ORF) and post-transcriptional regulatory element (PRE) [24] was generated as follows: pPB was digested with *Bst*EII and *Eco*RV and then self-ligated to delete nt 1406–2848, generating pεHCP. The ε sequence was additionally truncated by PCR-based mutagenesis, yielding pHCP. To generate a DHBV *wt* C protein construct containing the DHBV C ORF and HBV PRE, the DHBV C gene from pCMVDHBV (a gift from William Mason, Fox Chase Cancer Center) was cloned into pcDNA3 between the *Hin*dIII and *Eco*RV sites to yield pDC. The HBV PRE sequence was cloned into pDC to yield pDCP. Chimeric C protein variants were constructed by the PCR-derived recombination of HBV and DHBV C ORFs and the PCR-amplified fusion products cloned into the corresponding restriction sites of pHCP, yielding pHD192–262, pHD221–262, pHD192–220, pHD221–242, pHD242–262, pHHDH, and pHDHD. To generate pHCP145, a stop codon (TAG) was introduced at Thr146 (ACT) of HBV C protein by site-directed mutagenesis. To generate the assembly-deficient HBV variant [23], pHCP-R127Q, in which Arg127 (C**GC**) is modified to Gln (C**AG**) in pHCP by site-directed mutagenesis, was generated first; the *Hin*dIII- and *Bst*EII-digested DNA fragment from pHCP-R127Q, which contains the Arg127→Gln mutation, was then cloned into pHCP145, yielding the assembly-deficient pHCP145–R127Q variant. A C-deficient mutant that does not express C protein was generated by introducing a stop codon (**T**AA) at Glu8 (GAA) of the C protein by site-directed mutagenesis. This C-deficient mutant expresses pgRNA and all other HBV proteins except the C protein. To generate the C- deficient-RT-YMHA mutant, the *Eco*RI- and *Eco*RV-digested DNA fragment from a reverse transcriptase (RT) reaction deficient RT-YMHA mutant, wherein the conserved YMDD motif of the RT active site was modified to YMHA [27], was cloned into the C-deficient mutant. To further analyze the importance of Arg168, Arg169, Gln171, Ser172, and Arg175 residues (^167^R***RR***S***QS***PR***R*** ^175^ motif in HBV *vs* ^242^R***AG***S***PL***PR***S*** ^250^ motif in DHBV), A168R, G169R, P171Q, L172S, and S175R mutants were constructed in the HHDH C background by site-directed mutagenesis. To test *trans*-complementation of C-deficient-RT-YMHA or C-deficient mutants, a series of chimeric C proteins was used throughout the experiments for HBV encapsidation or HBV DNA replication. All constructs were sequenced to confirm the presence of specific mutations, and to ensure that no extraneous mutations were introduced during PCR.

### Anti-core Antibody Production

The amino-terminal 149 amino acids of HBV C protein DNA were PCR-amplified using primers HBV 181 (sense, 5′-*GTGCCTTGGATCC* CTTTGGGGCATGGAC-3′), and HBV 184 (antisense, 5′-*CGGTCCCGAAGCTT* AACAACAGTAGTTTCCGG-3′), which are specific for nt 1894–1908 and 2332–2349 of the HBV genome, respectively, and contain, respectively, *Bam*HI and *Hin*dIII sites for cloning. The PCR product was cloned into pET-21a, yielding PET-21a-C149-His. The PCR-derived DNA fragment was sequenced to ensure that there were no extraneous mutations. Sonicated lysates from *Escherichia coli* (BL21 DE3) that had been transformed with pET21a-C149 were centrifuged and the supernatants were subjected to nickel nitrilotriacetic acid (Ni-NTA) agarose bead column chromatography for purification. Purified C-149-His protein was injected into one rabbit. Preimmune serum was collected prior to immunization. After three subsequent immunizations, positive anti-serum against HBc protein was confirmed by Western blotting after sodium dodecyl sulfate-polyacrylamide gel electrophoresis (SDS-PAGE) on a 12% gel to detect HBV C protein or native agarose gel electrophoresis to detect core particles. This rabbit polyclonal anti-HBc antibody was used throughout the study to detect C protein variants (Figure S1). For detection of core particles, both polyclonal rabbit anti-HBc antibody (diluted 1∶1000; DAKO, Carpinteria, CA, USA) and our antibody (diluted 1∶1000) were used interchangeably (Figure S1).

### Cell Culture, Transfection, and Isolation of Core Particles

HuH7 hepatoma cells (Japan Health Sciences Foundation, Tokyo, Japan) were maintained as previously described [27]. For expression of chimeric C protein variants and assessment of their core particle formation, 8 µg of pHCP plasmid or various chimeric C protein constructs were transfected into HuH7 cells as previously described [27]. For analysis of pgRNA encapsidation or HBV DNA synthesis, 2 µg of C-deficient-RT-YMHA or C-deficient mutants and 6 µg of various chimeric C protein constructs were co-transfected into HuH7 cells as previously described [29]. 1 µg of the Renilla luciferase expression plasmid phRL-CMV (Promega, Madison, WI, USA) was co-transfected into HuH7 cells as a transfection control. pcDNA3.1 was used in transfections to equalize total DNA transfected. Cytoplasmic core particles were precipitated from lysates of transfected cells with 6.5% polyethylene glycol as previously described [27]. In brief, clarified lysate was adjusted with 10 mM MgCl_2_ and 8 mMCaCl_2_ solution, incubated overnight at 37°C with 20 U DNase I (Sigma) and 60 U micrococcal nuclease (Calbiochem) to remove the transfected plasmid DNA and unencapsidated RNA, and precipitated with 6.5% polyethylene glycol. Transfection experiments were repeated more than three times.

### RNase Protection Analysis (RPA)

To analyze encapsidated pgRNA, core particles were isolated as described above. pgRNA was extracted from core particles following digestion with proteinase K (100 µg/mL) and DNase I (20 U). To prepare riboprobe for RPA, nt 1805–2187 of the C-deficient mutant were cloned into pGEM3Zf(+) vector, generating pRPAFD-C-def. From this construct, 446 nt of radiolabeled anti-sense probe were synthesized *in vitro* using SP6 RNA polymerase with α-^32^P-UTP (specific activity, 800 Ci/mmol). The RPA procedure was performed using the manufacturer’s protocol (RPA II™, Ambion, Austin, TX, USA). Protected pgRNA was 369 nt in length. To discriminate encapsidated full-length pgRNA from spliced RNA, the spliced region containing probe, pRPA-PS, was also used [29]. The 470 nts of the HBV sequence was synthesized *in vitro* and the protected sequence, nt 2680–3092 of HBV sequence, was 413 nts long [29]. The relative levels of encapsidated pgRNA from isolated core particles were measured with the Image Gauge V4.0 program (Fujifilm, Tokyo, Japan).

### RNA Encapsidation Assay and Southern Blotting

To analyze pgRNA in core particles with chimeric C protein variants, pellets of core particles isolated from HuH7 cells co-transfected with the C-deficient-RT-YMHA mutant and various chimeric C protein constructs were dissolved in 15 µL Tris-acetate EDTA buffer and electrophoresed on 1% native agarose gels. Core particles were transferred to a nylon membrane and denatured with 0.2N NaOH *in situ* and neutralized. Nucleic acids from disrupted core particles were hybridized to a ^32^P-labeled random-primed probe specific for HBV sequence [29]. To analyze HBV DNA synthesis by Southern blotting, HBV DNA extracted from core particles was separated by agarose gel electrophoresis and hybridized to a ^32^P-labeled random-primed probe specific for HBV sequence [27]. The relative levels of pgRNA and HBV DNA isolated from core particles were measured with the Image Gauge V4.0 program.

### SDS-PAGE and Western Blotting

To analyze the C protein, total lysates were harvested and lysed in NP-40 containing lysis buffer (50 mM Tris-HCl [pH 8.0], 150 mM NaCl, 1% NP-40). The lysates were cleared by centrifugation and supernatants collected and 5% β-mercaptoethanol added; samples were then subjected to SDS-PAGE on 12% gels and the resolved proteins transferred to polyvinylidene fluoride (PVDF) membranes. These membranes were incubated with polyclonal rabbit anti-HBc antibody, monoclonal mouse anti-tubulin (diluted 1∶1000; Calbiochem, San Diego, CA, USA), or polyclonal rabbit anti-luciferase (diluted 1∶500; Santa Cruz Biotechnology, Santa Cruz, CA, USA) antibodies. Immunoreactive bands were visualized by a horseradish-peroxidase conjugated secondary antibody (DAKO) using enhanced chemiluminescence (Amersham, Piscataway, NJ, USA). Western blot analysis of core particles was performed as previously described [27], [29]. Isolated core particles were electrophoresed on a 1% native agarose gel and resolved core particles transferred to PVDF membranes. Immunoblotting was performed using polyclonal rabbit anti-HBc antibody (diluted 1∶1000). Horseradish-peroxidase conjugated anti-rabbit secondary antibody and enhanced chemiluminescence were employed to visualize HBV core particles.

### PCR to Detect HBV DNA from Spliced RNA

HBV DNA was extracted from core particles isolated from HuH7 cells co-transfected with the C-deficient mutant and various C variant constructs. PCR was performed using primers HBV 155 (sense 5′-CTACTGTGGAGTTACTCTCG-3′) and HBV 8, (antisense 5′- CACGATGCTGTACAGACTTG -3′), which correspond to position nt 1935–1954 and nt 706–725 of the HBV genome, respectively. PCR amplified products were separated by agarose gel electrophoresis, gel-purified, and sequenced.

## Supporting Information

Figure S1 **Detection of C protein and core particles by rabbit polyclonal HBc antibodies.** To examine home-made rabbit polyclonal anti-HBc antibody (diluted 1∶1000; upper panels) and polyclonal rabbit anti-HBc antibody (diluted 1∶1000; DAKO, Carpinteria, CA, USA; lower panels), Western blotting after SDS-PAGE on a 12% gel to detect HBV C protein (left panels) or native agarose gel electrophoresis to detect core particles (right panels) was performed on lysates from HuH7 cells transfected with HBV *wt*, pHCP, pHCP145, pHCP145-R127Q, pHDHD, or pHHDH variants, as described for Figure 1B.
(TIF)

Figure S2 **RPA using spliced-out region RPA-PS probe.** (A) RPA to discriminate encapsidated full-length pgRNA and spliced RNA. To detect the pgRNA encapsidated by chimeric C protein variants, the C-deficient-RT-YMHA mutant and the C protein chimeras were co-transfected into HuH7 cells as described for Figure 7. RPA was performed as described for Figure 2D using spliced-region probe. The 470 nts of the HBV sequence was synthesized *in vitro* and the protected sequence, nt 2680-3092 of HBV sequence, was 413 nts long [29]. Transfection experiments were repeated five times. Relative levels of encapsidated pgRNA were measured with the Fujifilm Image Gauge V4.0 program. The data represent the mean ± SD from four independent experiments. * p<0.001, ** p<0.01 (n = 5).
(TIF)
