# Supplementary figures and images for: C-Terminal Substitution of HBV Core Proteins with Those from DHBV Reveals That Arginine-Rich 167RRRSQSPRR175 Domain Is Critical for HBV Replication
Source: PLoS One. 2012 Jul 20;7(7):e41087. doi: 10.1371/journal.pone.0041087 (PMC3401125; doi:10.1371/journal.pone.0041087)

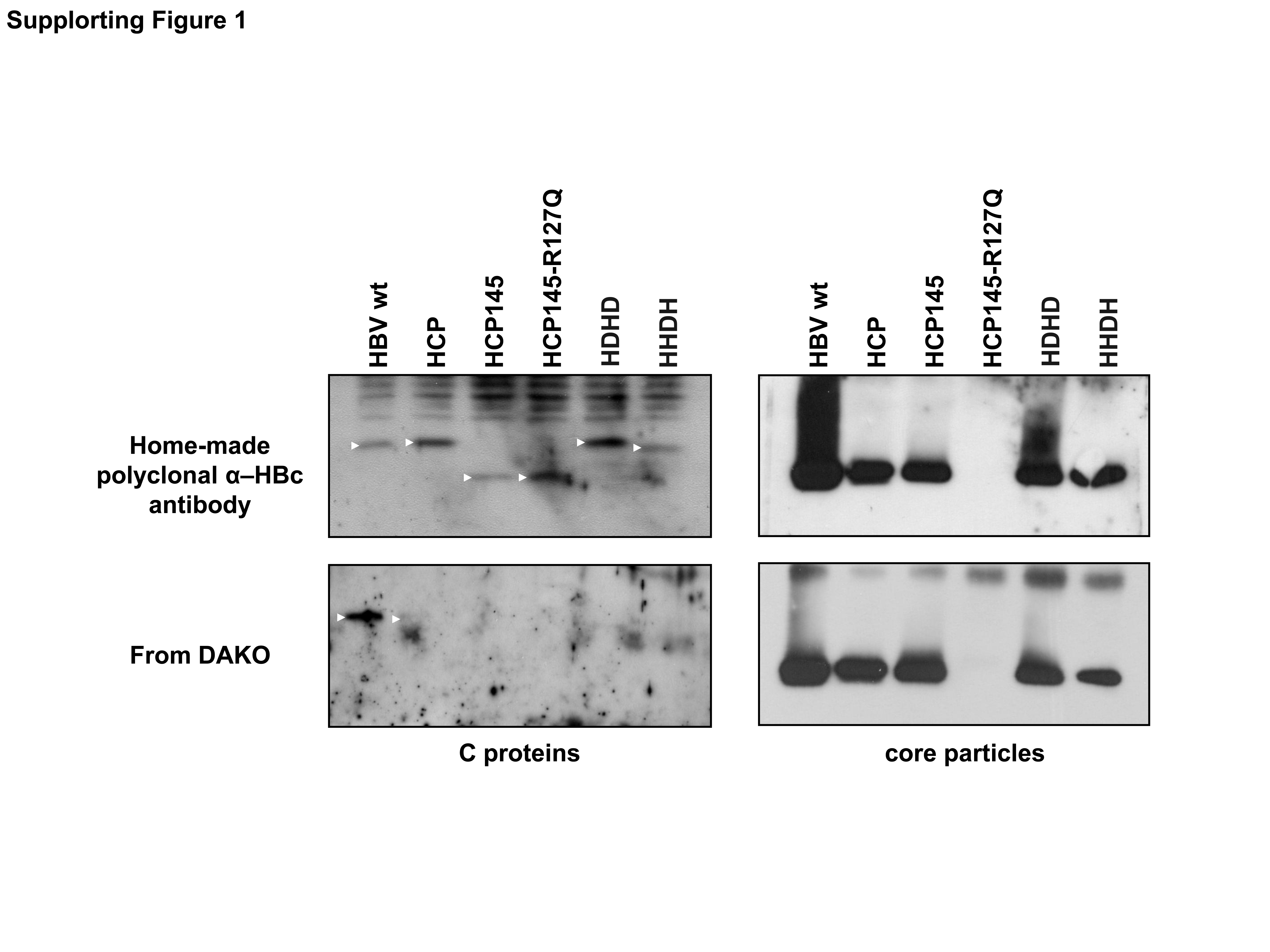

Supplement: Figure S1 — Detection of C protein and core particles by rabbit polyclonal HBc antibodies. To examine home-made rabbit polyclonal anti-HBc antibody (diluted 1∶1000; upper panels) and polyclonal rabbit anti-HBc antibody (diluted 1∶1000; DAKO, Carpinteria, CA, USA; lower panels), Western blotting after SDS-PAGE on a 12% gel to detect HBV C protein (left panels) or native agarose gel electrophoresis to detect core particles (right panels) was performed on lysates from HuH7 cells transfected with HBV wt, pHCP, pHCP145, pHCP145-R127Q, pHDHD, or pHHDH variants, as described for Figure 1B. (TIF) [file pone.0041087.s001.tif]

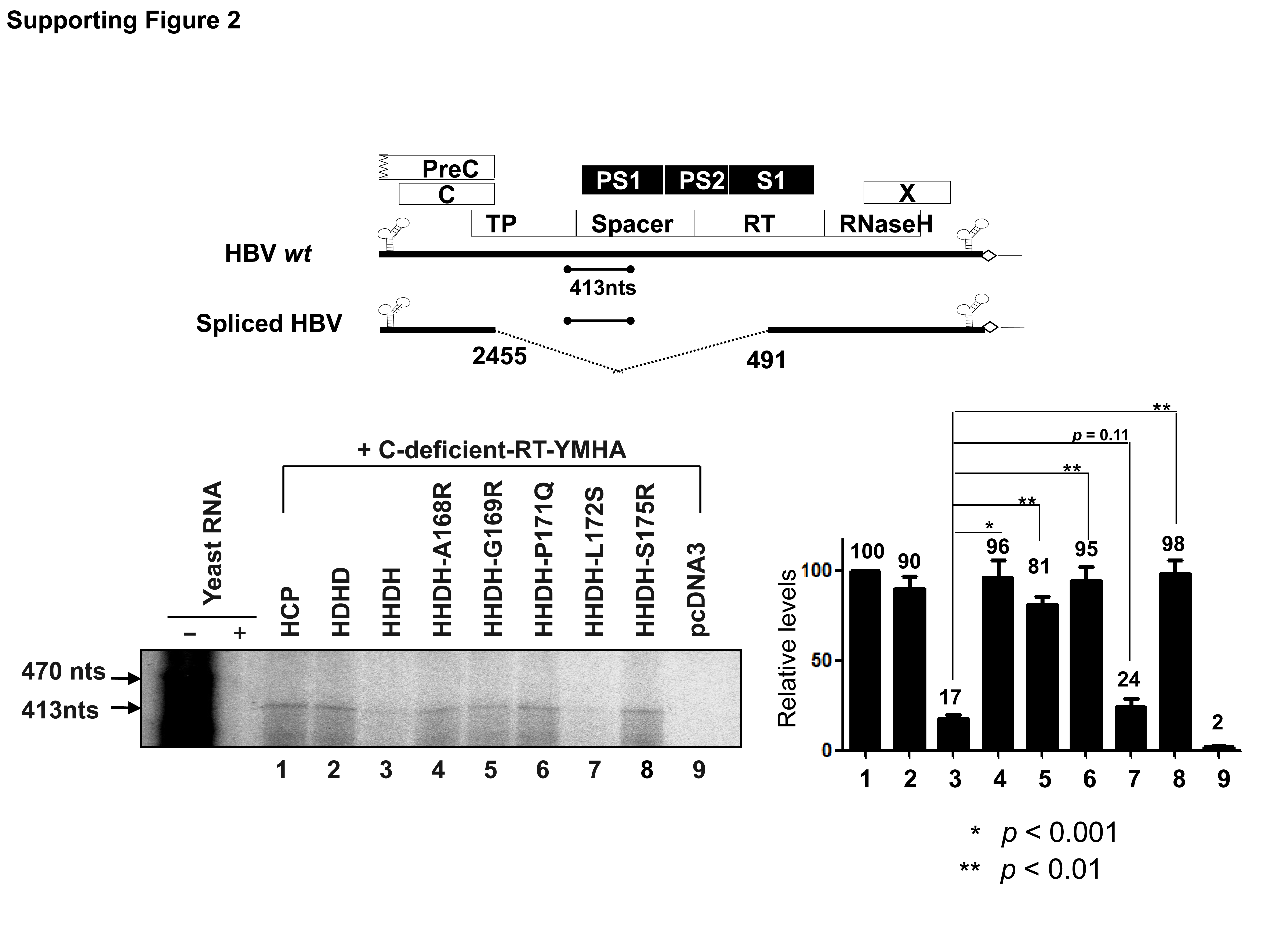

Supplement: Figure S2 — RPA using spliced-out region RPA-PS probe. (A) RPA to discriminate encapsidated full-length pgRNA and spliced RNA. To detect the pgRNA encapsidated by chimeric C protein variants, the C-deficient-RT-YMHA mutant and the C protein chimeras were co-transfected into HuH7 cells as described for Figure 7. RPA was performed as described for Figure 2D using spliced-region probe. The 470 nts of the HBV sequence was synthesized in vitro and the protected sequence, nt 2680-3092 of HBV sequence, was 413 nts long [29]. Transfection experiments were repeated five times. Relative levels of encapsidated pgRNA were measured with the Fujifilm Image Gauge V4.0 program. The data represent the mean ± SD from four independent experiments. * p<0.001, ** p<0.01 (n = 5). (TIF) [file pone.0041087.s002.tif]
